# A voxel-based morphometry investigation of brain structure variations in late-life depression with insomnia

**DOI:** 10.3389/fpsyt.2023.1201256

**Published:** 2023-05-18

**Authors:** Heng Shao, Na Li, Meiling Chen, Jie Zhang, Hui Chen, Minjun Zhao, Jingjing Yang, Jian Xia

**Affiliations:** ^1^Department of Psychiatry, First Affiliated Hospital of Kunming Medical University, Kunming, China; ^2^Department of Clinical Psychology, The First People’s Hospital of Yunnan Province, Kunming, China; ^3^The Affiliated Hospital of Kunming University of Science and Technology, Kunming, China; ^4^Department of MRI, The First People’s Hospital of Yunnan Province, Kunming, China; ^5^Department of Geriatrics, The First People’s Hospital of Yunnan Province, Kunming, China; ^6^School of Medicine, Kunming University of Science and Technology, Kunming, China

**Keywords:** voxel-based morphometry, late-life depression, insomnia, anxiety, cerebellum

## Abstract

**Background:**

Late-life depression (LLD) is linked to various medical conditions and influenced by aging-related processes. Sleep disturbances and insomnia symptoms may be early indicators or risk factors for depression. Neuroimaging studies have attempted to understand the neural mechanisms underlying LLD, focusing on different brain networks. This study aims to further delineate discriminative brain structural profiles for LLD with insomnia using MRI.

**Methods:**

We analyzed 24 cases in the LLD with insomnia group, 26 cases in the LLD group, and 26 in the healthy control (HC) group. Patients were evaluated using the Hamilton Depression Rating Scale (HAMD-17), Hamilton Anxiety Rating Scale (HAMA), Mini-Mental State Examination (MMSE), and Pittsburgh Sleep Quality Index (PSQI). Structural MRI data were gathered and analyzed using voxel-based morphometry (VBM) to identify differences in gray matter volume (GMV) among the groups. Correlation analyses were conducted to explore the relationships between GMV and clinical characteristics.

**Results:**

Significant difference in sex distribution was observed across the groups (*p* = 0.029). However, no significant differences were detected in age and MMSE scores among the groups. LLD with insomnia group exhibited significantly higher HAMA (*p* = 0.041) and PSQI scores (*p* < 0.05) compared to the LLD group. ANOVA identified significant difference in GMV of anterior lobe of cerebellum (peak MNI coordinate: *x* = 52, *y* = −40, *z* = −30) among HC, LLD, and LLD with insomnia. Post-hoc two-sample *t*-tests revealed that the significant difference in GMV was only found between the LLD group and the HC group (*p* < 0.05). The mean GMV in the cerebellum was positively correlated with HAMA scale in LLD patients (*r* = 0.47, *p* < 0.05).

**Conclusion:**

There is significant difference in GMV in the LLD group, the association between late-life depression and insomnia may be linked to anxiety. This study provides insights into the discriminative brain structural profiles of LLD and LLD with insomnia, advancing the understanding of the underlying neural mechanisms and potential targets for intervention.

## Introduction

The aging population is a growing global issue, with the United Nations forecasting that by 2050, one in six people worldwide will be over 65 years old (1). Mental disorders, such as depression and anxiety, are a major contributor to the global burden of disease in the elderly (2). Approximately 16% of older adults living in the community may experience clinically significant symptoms of depression, with an incidence of 29% in Europe (3). In Southeast Asia, the overall prevalence of late-life depression (LLD) is estimated to be 42.0%, and in China, the prevalence is approximately 20% (4, 5). LLD is characterized by persistent feelings of sadness, hopelessness, a lack of interest in activities, executive dysfunction, impaired cognitive function, fatigue and insomnia (6–8). Changes in sleep patterns are a normal part of aging, however, older adults may have difficulty falling asleep and staying asleep due to awakenings, and tend to fall asleep earlier and wake up earlier than younger adults (9). Several studies have indicated that insomnia symptoms, excessive daytime sleepiness, and drug use independently increase the risk of subsequent depression in older adults. Sleep disturbance and long-term use of sleeping pills may be early indicators or potentially risk factors for depression (10–13).

LLD is linked to a variety of medical conditions and is thought to be influenced by aging-related processes (14). Potential causes of LLD include vascular damage, chronic inflammation, and dysfunction in brain networks (15–18). Studies using neuroimaging techniques have attempted to understand the neural mechanisms underlying LLD, with a focus on networks such as the executive control network (ECN), default mode network (DMN), cognitive control network (CCN), affective prelimbic network, and corticostriatal circuits (19). Functional connectivity (FC) impairments within or between the frontoparietal, dorsal attention, visual networks (20), as well as subcortical networks of thalamus, basal ganglia, and ventral striatum (21, 22) have been observed in individuals with LLD. Additionally, gray matter reductions have been observed in the anterior cingulate cortex, dorsolateral, and dorsomedial prefrontal cortex in LLD patients (21). Insomnia has been also linked to dysfunction in both intra-hemispheric and inter-hemispheric connectivity (22). Studies have identified increased connectivity in certain brain regions of the DMN and frontoparietal network (23), bilateral hippocampus, and left middle frontal gyrus (24, 25). However, the current existing neuroimaging findings about LLD are lack of consistency since other studies also found reductions in connectivity in areas such as the anterior cingulate cortex, orbital frontal cortex, hippocampus, caudate, and putamen (26). Although several studies have identified structural and functional abnormalities in LLD, the specific brain structural changes associated with LLD with insomnia remain unclear. To further delineate discriminative brain structural profiles for LLD with insomnia, structural MRI were acquired. We employed voxel-based morphometry (VBM) to analyze gray matter volume (GMV) in LLD patients with insomnia. Our hypothesis is that affective brain regions in LLD with insomnia may exhibit different GMV patterns.

## Methods

### Participants

In this retrospective study, we scrutinized cases obtained from the Clinical Psychology and Geriatrics departments at the First People’s Hospital of Yunnan Province spanning from January 2021 to October 2022. All included patients were aged over 60 years and right-handed. Additionally, patients underwent Hamilton Depression Rating Scale (HAMD-17), Hamilton Anxiety Rating Scale (HAMA), and Mini-Mental State Examination (MMSE) scoring. Patients with LLD were included based on the DSM-V diagnostic criteria and HAMD-17 scores ≥17. Sleep assessments were conducted using the Pittsburgh Sleep Quality Index (PSQI) criteria, with insomnia defined as PSQI scores ≥5. Scores were based on sleep quality, sleep onset latency, sleep duration, sleep efficiency, sleep disturbances, hypnotic medication use, and daytime dysfunction dimensions (48). Group A comprised patients with LLD and insomnia, Group B consisted of patients with LLD, and Group C served as the healthy control (HC) group with HAMD-17 scores below 17 and PSQI scores below 5.

Exclusion criteria included: severe central nervous system diseases such as ischemic stroke, cerebral hemorrhage, or subarachnoid hemorrhage, accompanied by Parkinson’s disease or multiple sclerosis; abnormal brain white matter caused by carbon monoxide poisoning, hydrocephalus, or hypoxic brain injury; respiratory diseases affecting sleep, like cough variant asthma and obstructive sleep apnea hypopnea syndrome (OSAHS); severe cardiovascular, digestive, and urinary system diseases; long-term alcohol consumption exceeding 14 drinks per week; a history of bipolar disorder, personality disorder, or schizophrenia; and inability to undergo a brain MRI examination.

Initially, 138 patients met these criteria; however, due to factors such as MRI artifacts affecting the magnetic resonance data processing, only 76 cases were ultimately retained. The final sample included 24 cases in the LLD with insomnia group, 26 in the LLD group, and 26 in the healthy control group.

### Structural MRI data collection

The structural MRI data were acquired using a SIEMENS 1.5-T (Magnetom Skyra, Siemens-Healthcare) scanner with a standard head coil. Image acquisition was performed in the Department of Magnetic Resonance Imaging, The First People’s Hospital of Yunnan Province. For each subject, a high-resolution Fluid Attenuated Inversion Recovery (FLAIR) 3D sequence has been acquired with parameters of Slice Thickness 0.7 mm without gap, repetition time (RT) = 2,200 ms, echo time (ET) = 5.88 ms, flip angle (FA) = 25°, covering 118 axial slices with an in-plane resolution of 0.69 mm × 0.69 mm.

### Voxel-based morphometry analysis

The high-spatial-resolution structural MRI data were processed using the VBM Toolbox (VBM8)^1^ in the statistical parametric mapping software (SPM 8).^2^ First, any images with artifacts were removed. Next, the images were segmented into gray matter (GM), white matter (WM), and cerebrospinal fluid (CSF) using the default settings in VBM8, and then normalized to the Montreal Neurologic Institute (MNI) space and modulated. After the quality of the segmentation was confirmed, the segmented GM images were smoothed using an 8-mm full-width-at-half-maximum Gaussian kernel for group statistical comparisons. A one-way analysis of variance (ANOVA) with sex, anxiety level, and total brain volume as a covariates was used to identify any significant differences in GMV between LLD with insomnia, LLD, and HC groups. The significant level was determined using Alphasim corrected method with *p* < 0.05 at the voxel-level threshold of *p* < 0.005.

### Statistical data and correlation analysis

A one-way ANOVA was initially employed to detect differences in demographic and clinical features. Subsequently, post-hoc two-sample *t*-tests were utilized to ascertain between-group differences for all indices. For gender comparisons, a Pearson chi-squared test was conducted. Two-sample *t*-tests were applied to examine other variables.

In order to establish if there was a relationship between changes in GMV and clinical features, correlation analyses were carried out with a significance level set at *p* < 0.05, without correction.

## Results

### Demographics and clinical characteristics

The details of demographics and clinical characteristics of all the subjects are shown in Table 1. A significant difference in sex distribution was observed across the groups (*p* = 0.029), with a higher proportion of females in the LLD with insomnia and LLD groups compared to the HC group. However, no significant differences were detected in age and MMSE scores among the groups. Both the LLD with insomnia (*p* < 0.05) and LLD (*p* < 0.05) groups demonstrated significantly higher HAMD and HAMA scores compared to the HC group. Furthermore, the LLD with insomnia group exhibited significantly higher HAMA (*p* = 0.041) and PSQI scores (*p* < 0.05) compared to the LLD group.

**Table 1:** Demographics and clinical characteristics of the subjects used in this study.

	LLD-INS (*n* = 24)	LLD (*n* = 26)	HC (*n* = 26)	*F* value	HC vs. LLD-INS	HC vs. LLD	LLD vs. LLD-INS
Sex (F/M)	15/9	18/8	9/17	*F* = 3.73 (*p* = 0.029)	*p* = 0.05	*p* = 0.012	*p* = 0.62
Age (years)	65.21 ± 7.13	65.42 ± 5.52	64.58 ± 4.95	*F* = 0.14 (*p* = 0.87)	*p* = 0.72	*p* = 0.56	*p* = 0.91
HAMD scores	21.33 ± 5.29	18.96 ± 5.06	6.23 ± 3.35	*F* = 78.38 (*p* < 10^−18^)	*p* = 10^−15^	*p* = 10^−13^	*p* = 0.11
HAMA scores	16.83 ± 5.51	13.15 ± 6.77	5.31 ± 2.63	*F* = 31.64 (*p* < 10^−9^)	*p* = 10^−11^	*p* = 10^−15^	*p* = 0.041
PSQI	16.38 ± 2.65	6.88 ± 2.45	3.77 ± 1.03	*F* = 229.46 (*p* < 10^−31^)	*p* < 10^−26^	*p* < 10^−6^	*p* = 10^−16^
MMSE	25.88 ± 3.01	25.19 ± 4.6	27.04 ± 1.84	*F* = 2.01 (*p* = 0.14)	*p* = 0.1	*p* = 0.064	*p* = 0.54

One-way analysis of variance (ANOVA) was first used to identify differences in demographics and clinical characteristics. Post-hoc two-sample *t*-tests were further used to determine the between group differences in all the indices. A Pearson chi-squared test was used for gender comparison. Two-sample *t*-tests were used for other variables. LLD-INS, Late-life depression with insomnia; LLD, Late-life depression; HC, Health Control.

### GMV differences and correlation results

**Figure 1:**
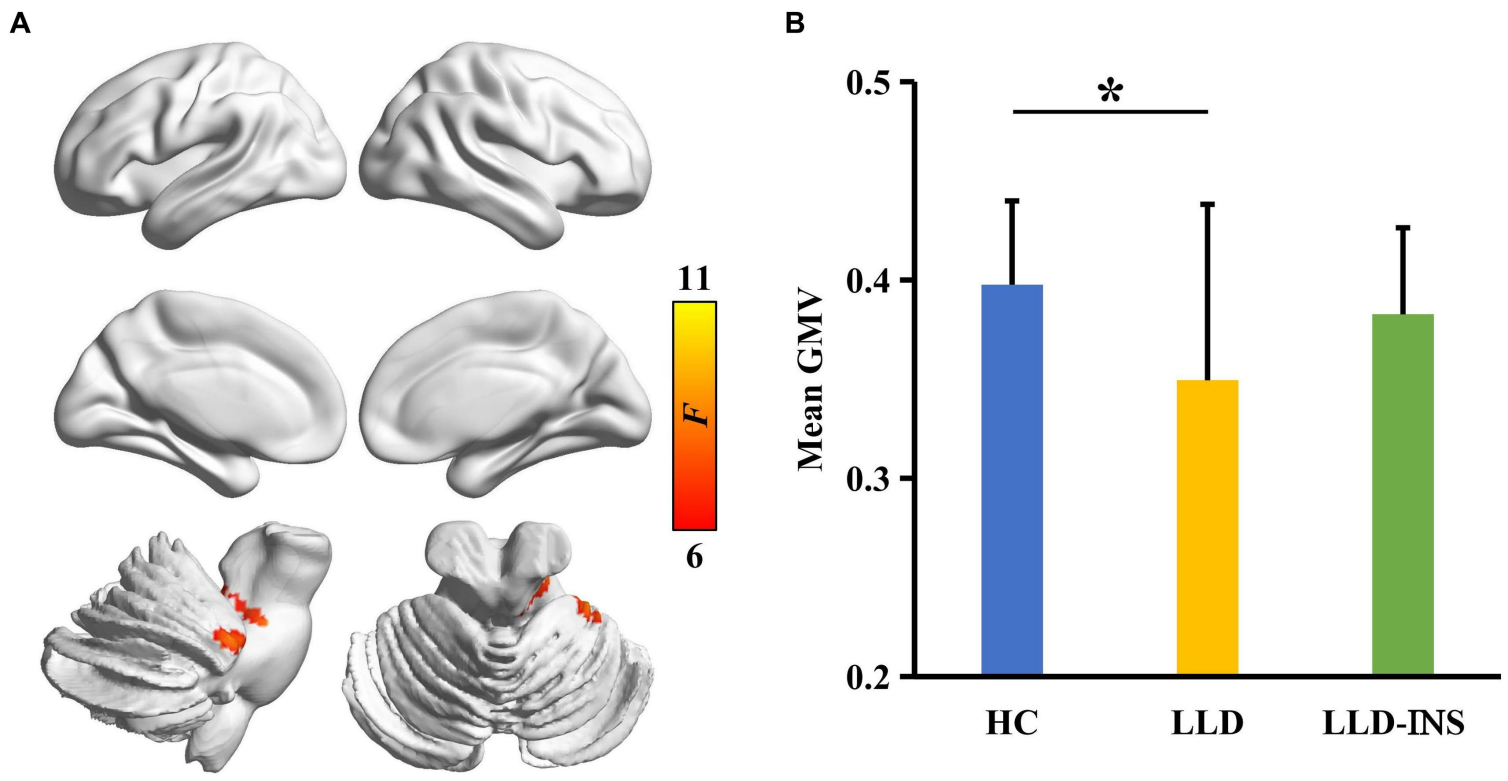
**(A)** Analysis of variance (ANOVA) with sex as a covariate was performed to identify gray matter volume (GMV) differences across LLD with insomnia, LLD, and HC groups, revealing a significant difference in GMV within the cerebellum. **(B)** Post-hoc two-sample t-tests were conducted between each pair of groups, identifying a significant difference in GMV between the LLD and HC groups.

**Figure 2:**
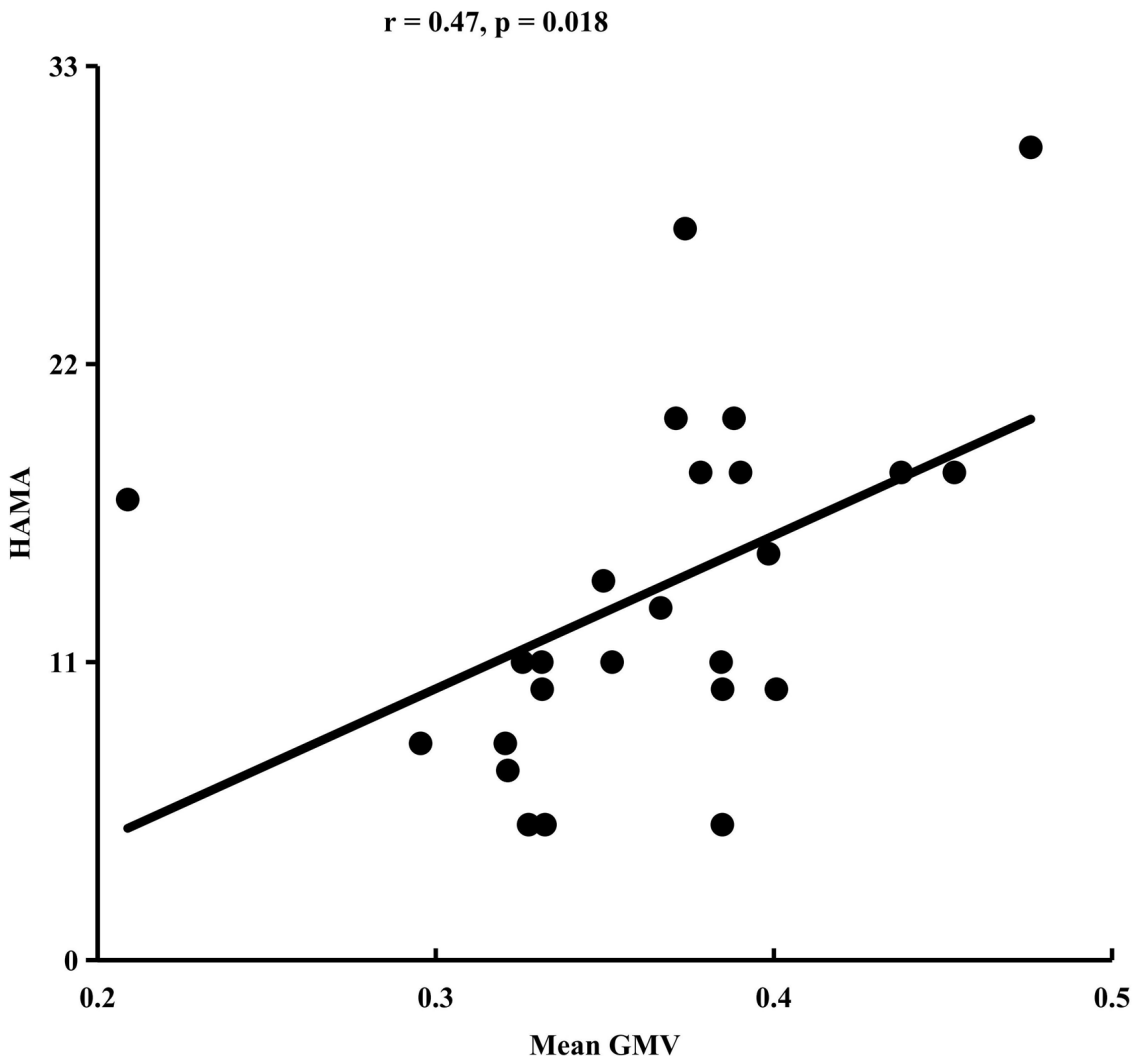
Gray matter volume (GMV) in the cerebellum correlates with HAMA scores in late-life depression (LLD).

ANOVA identified significant difference in GMV of anterior lobe of cerebellum (peak MNI coordinate: *x* = 52, *y* = −40, *z* = −30) among HC, LLD, and LLD with insomnia (Figure 1A). Post-hoc two-sample *t*-tests revealed that the significant difference in GMV was only found between the LLD group and the HC group (*p* < 0.05) (Figure 1B). The mean GMV in the cerebellum was positively correlated with HAMA scale in LLD patients (*r* = 0.47, *p* < 0.05) (Figure 2).

## Discussion

Our results have shown that LLD patients with insomnia have higher HAMA scores than the LLD group. This is in line with previous research that has found high rates of comorbid anxiety disorders (83.62%) and insomnia (74.52%) in LLD patients. Vice versa, insomnia serves as a risk factor for the development of both depression (27). Additionally, the large-sample study has revealed that the average cost of the depression-anxiety group was higher than that of the non-anxiety group (28). One of the unique aspects of insomnia in older adults is that it often co-occurs with other chronic medical conditions, such as heart disease, stroke and diabetes (29). In older adults, somatic symptoms associated with these conditions may contribute to anxiety, which in turn may aggravate insomnia or interact with each other (30, 31). The HAMD-17 scale, frequently employed for assessing depression, may lack the sensitivity required to differentiate between somatic symptoms and the impacts of insomnia (30). Therefore, when evaluating insomnia in older adults, it is important to consider both anxiety symptoms and chronic conditions to gain a comprehensive understanding of underlying causes and potential treatment options. It is also important to be aware that when sedative drugs are used in the elderly, they may increase the risk of all-cause dementia and Alzheimer’s disease (AD) (32).

The most significant difference in GMV between the three groups was found in cerebellum. The cerebellum, a vital motor organ, plays a crucial role in maintaining balance and controlling voluntary movement, particularly in coordinating skilled visual movement with the cerebral cortex (33). However, recent researches have uncovered that cerebellum is far more extensive and complex than previously reported (34). The cerebellum is involved in a diverse range of learning and timing processes, and different sub-regions of the cerebellum utilize different forms of plasticity. In addition to the motor functions, it also plays a vital role in regulating cognitive processes, learning and emotions. The cerebellum maintains extensive connections with various brain structures, including the reticular system, hypothalamus, limbic system, and neocortical association areas (35). Moreover, the cerebellum acts as brain connectome and neural circuits, consolidating information originating from nuclei such as the subthalamic nucleus within the basal ganglia and the dentate nucleus situated in the cerebellum itself. This information is then relayed to the cerebral cortex, establishing cohesive networks that orchestrate motor, cognitive, and emotional processes (36, 37). Damage to the cerebellum can cause motor symptoms such as dysmetria, dysarthria, and ataxia, as well as cognitive and affective symptoms, referred as cerebellar cognitive affective syndrome (38). Cerebellar dysfunction also disrupts normal sleep–wake cycles and causes various sleep disorders such as insomnia, excessive daytime sleepiness, rapid eye movement (REM) behavioral disturbances, and sleep apnea (39). Studies have shown a strong correlation between sleep disturbances and cerebellar pathology, possibly due to the close connections between the cerebellum and cerebral cortex. Sleep is crucial for cognitive processes and the interaction between the cerebral cortex and cerebellum during both wakefulness and sleep, contributes to memory consolidation (40).

The brain has complex connectivities, and different brain regions interacting in intricate and dynamic ways are responsible for different functions. The specific pattern of connections plays a crucial role in determining the onset, manifestation, and progression of various brain disorders (41). Understanding the underlying connectivity patterns in the brain may help to better understand the mechanisms that drive these disorders and identify potential targets for treatment. Functional MRI studies have revealed alterations in functional networks within specific brain regions in patients with persistent insomnia, such as changes in activity in the cingulate and right para-hippocampus, cerebellum, and superior frontal gyrus (42). Additionally, anxious patients may have decreased connectivity within hippocampus and amygdala and increased or decreased connectivity with cerebellum (43, 44). A previous study has reported that individuals with poor subjective sleep quality tend to have reduced cognitive function and more severe depressive symptoms (45). Our results indicate that there is a positive correlation between mean cerebellar GMV and scores on the HAMA scale. Previous researches have also demonstrated that anxiety lead to changes in local gray matter volume, which is positively correlated with the volumes of the dorsomedial prefrontal cortex and the anterior cingulate cortex (46, 47). However, this does not establish a causal link between gray matter volume and anxiety. It is important to note that this is a bidirectional relationship, where insomnia may lead to LLD, and vice versa.

### Limitation

Firstly, due to the nature of this observational study, it is incapable of establishing causality, and the findings merely indicate correlations. Secondly, the limited sample size restricts the generalizability of the results and the study’s capacity to discern potential differences. Consequently, cautious interpretation of the results is crucial, and alternative explanations for the findings should be considered. It is also worth noting that VBM is a relatively nascent technique, with ongoing research dedicated to refining methodologies and enhancing accuracy. Moreover, VBM analyses are influenced by the age of the study population, and the understanding of brain structural changes in elderly populations remains limited.

## Conclusion

The association between late-life depression and insomnia may be linked to anxiety, which could stem from age-related or somatic diseases in the elderly. When addressing sleep concerns in this population, it is essential to also consider the potential presence of chronic diseases or somatic symptoms. The cerebellum’s role in late-life depression should not be overlooked, as it may offer new perspectives and research directions for understanding and addressing depression in older adults with insomnia.

## Data availability statement

The original contributions presented in the study are included in the article/supplementary material, further inquiries can be directed to the corresponding author.

## Ethics statement

The studies involving human participants were reviewed and approved by the institutional review board of the First People’s Hospital of Yunnan Province. The patients/participants provided their written informed consent to participate in this study.

## Author contributions

HS, NL, MC, and HC performed the conceptualization, methodology, drafting of the original manuscript, supervision, and validation. JZ collected and processed the MRI data. MZ, JY, and JX handled methodology, statistical analysis, data management, and review. HS and NL reviewed, edited, and proofreaded. All authors contributed to this article and approved the submitted version.

## Funding

This study was supported by the study of relationship between the expression of vascular growth factor and the prognosis of type 2 diabetes mellitus combined with major depressive disorder (Grant No. 202001AY070001-035); Yunnan Clinical Research Center for Mental Diseases (Grant No. 0105679005).

## Conflict of interest

The authors declare that the research was conducted in the absence of any commercial or financial relationships that could be construed as a potential conflict of interest.

## Publisher’s note

All claims expressed in this article are solely those of the authors and do not necessarily represent those of their affiliated organizations, or those of the publisher, the editors and the reviewers. Any product that may be evaluated in this article, or claim that may be made by its manufacturer, is not guaranteed or endorsed by the publisher.

## Footnotes

^1^ http://dbm.neuro.uni-jena.de/vbm8/

^2^ https://www.fil.ion.ucl.ac.uk/spm/software/spm8/
